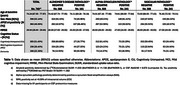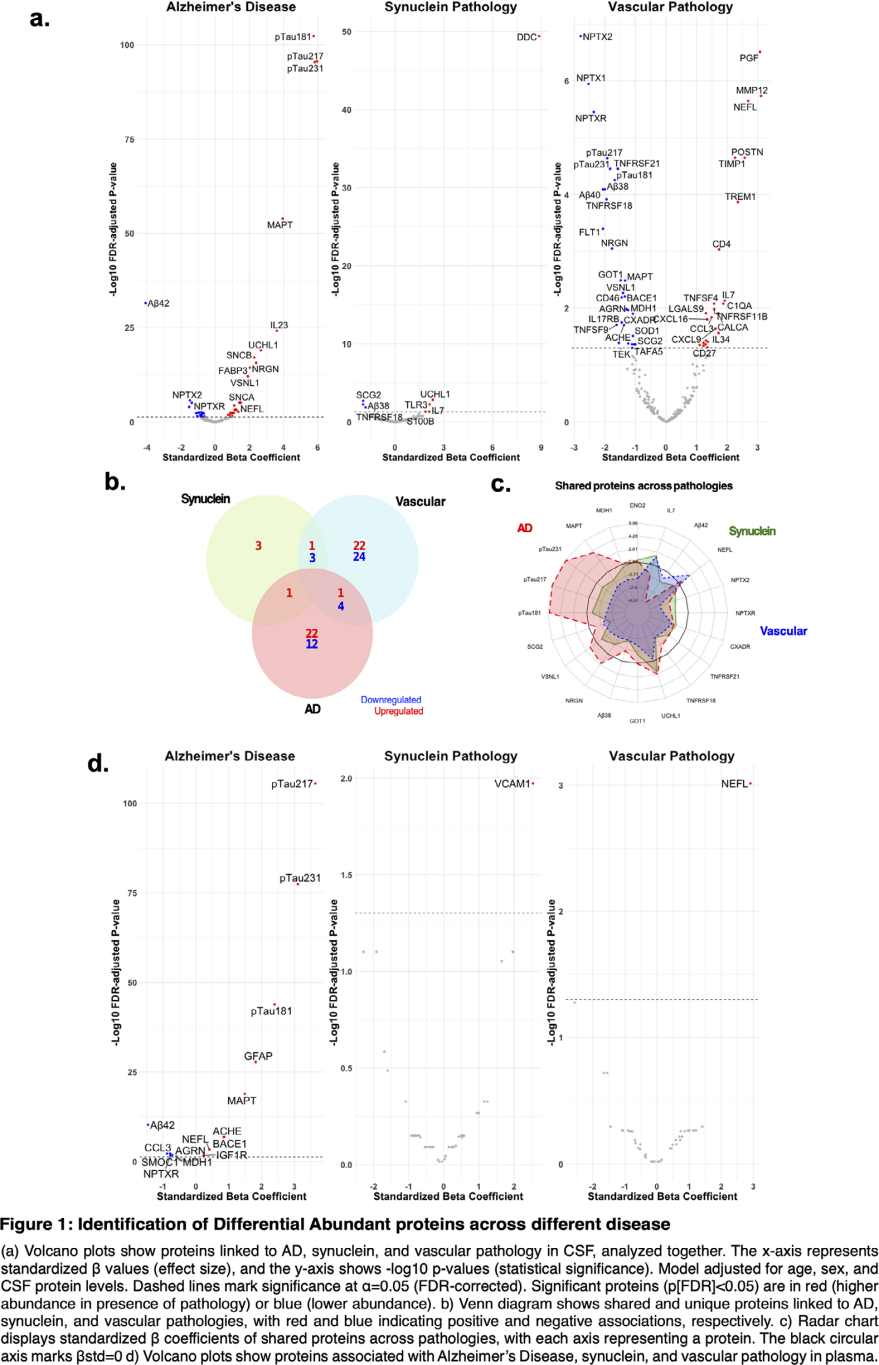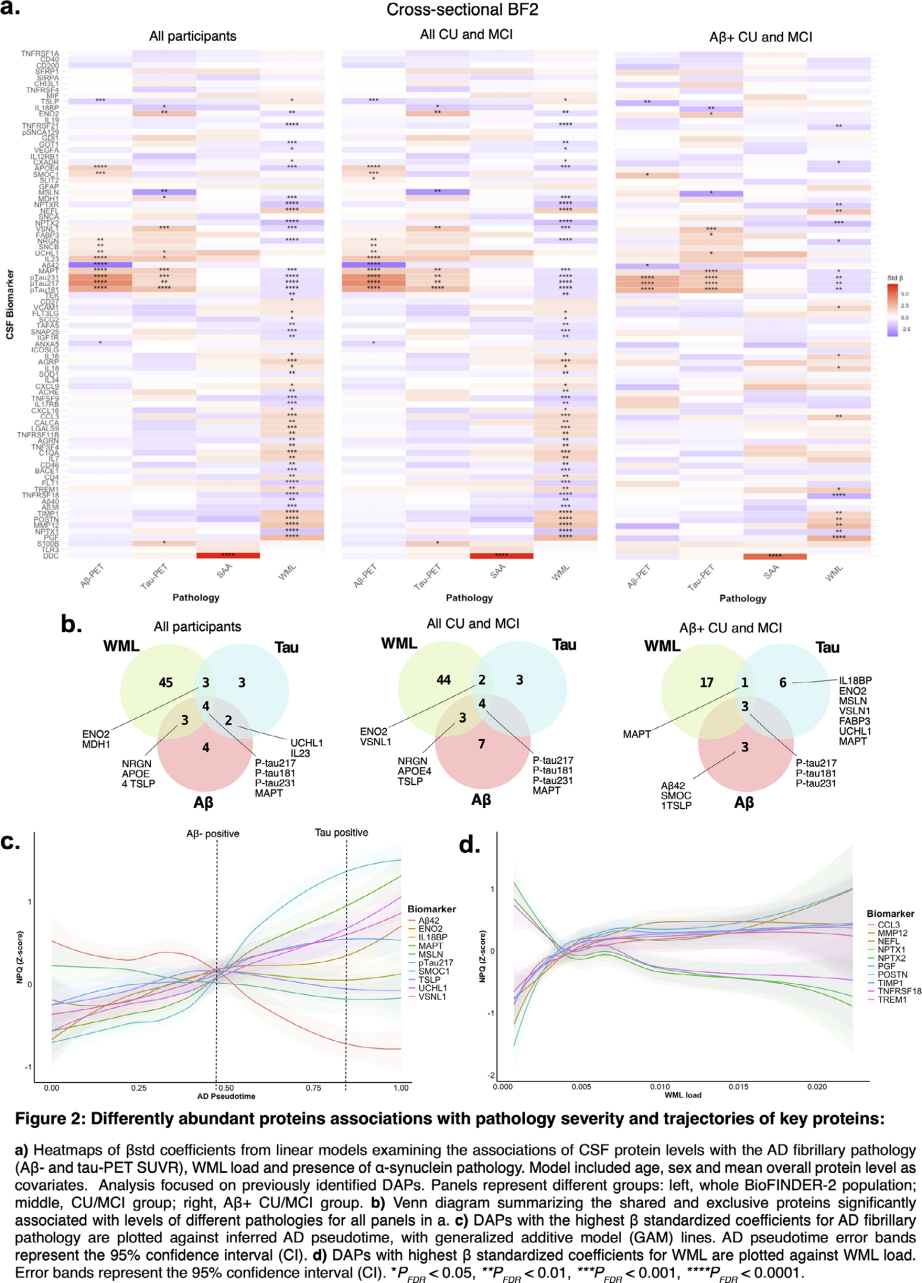# Identification of Differentially Abundant Proteins in Alzheimer's, Synuclein, and Vascular Pathologies Using the NULISA proteomics platform

**DOI:** 10.1002/alz70856_099280

**Published:** 2025-12-24

**Authors:** Anna Orduña Dolado, Andrea Benedet, Alexa Pichet Binette, Ines Hristovska, Ilaria Pola, Kübra Tan, Wiebke Traichel, Gemma Salvadó, Shorena Janelidze, Sebastian Palmqvist, Niklas Mattsson‐Carlgren, Nicholas Ashton, Oskar Hansson

**Affiliations:** ^1^ Clinical Memory Research Unit, Department of Clinical Sciences, Lund University, Lund, Sweden; ^2^ Institute of Neuroscienace and Physiology, University of Gothenburg, Mölndal, Västra Götaland, Sweden; ^3^ Clinical Memory Research Unit, Department of Clinical Sciences Malmö, Faculty of Medicine, Lund University, Lund, Sweden; ^4^ Department of Psychiatry and Neurochemistry, Institute of Neuroscience and Physiology, The Sahlgrenska Academy, University of Gothenburg, Mölndal, Sweden; ^5^ Barcelonaβeta Brain Research Center (BBRC), Pasqual Maragall Foundation, Barcelona, Spain; ^6^ Memory Clinic, Skåne University Hospital, Malmö, Skåne, Sweden; ^7^ Clinical Memory Research Unit, Department of Clinical Sciences Malmö, Lund University, Lund, Sweden; ^8^ Clinical Memory Research Unit, Lund University, Malmö, Skåne, Sweden; ^9^ Wallenberg Center for Molecular Medicine, Lund University, Lund, Sweden; ^10^ Banner Alzheimer's Institute, Phoenix, AZ, USA; ^11^ King's College London, Institute of Psychiatry, Psychology & Neuroscience, Maurice Wohl Clinical Neuroscience Institute, London, United Kingdom; ^12^ NIHR Biomedical Research Centre for Mental Health and Biomedical Research Unit for Dementia at South London and Maudsley NHS Foundation, London, United Kingdom; ^13^ Centre for Age‐Related Medicine, Stavanger University Hospital, Stavanger, Norway

## Abstract

**Background:**

The revised Alzheimer's Association criteria for Alzheimer's Disease (AD) diagnosis highlights the importance of co‐pathologies, such as synucleinopathy and vascular pathology, alongside amyloid‐β (Aβ) and tau. This study used the automated NULISA platform to differentiate AD, α‐synuclein, and vascular pathology in cerebrospinal fluid (CSF) and plasma.

**Method:**

We included 749 participants from the BioFINDER‐1 and BioFINDER‐2 cohorts (Table‐1). CSF was analyzed using the NULISA CNS and Inflammation panels (324 proteins), while plasma included only the CNS panel (*n* = 125). Differentially abundant proteins (DAPs) were identified using a linear model including including presence of all pathologies (binary measures of AD pathology, α‐synuclein and vascular pathology) and adjusting for age, sex, and average protein levels. In BioFINDER‐2 subcohort (*n* = 641), DAPs were then evaluated for associations with continuous Aβ‐PET, tau‐PET, white matter lesions (WML) load, and presence of α‐synuclein pathology, all pathologies and covariates combined in one model. Protein trajectories were plotted along the AD pseudotime (as a measure of AD pathology) and WML load. AD pseudotime was calculated using plasma *p*‐tau217 and tau‐PET SUVR from three meta‐ROI.

**Result:**

We identified 84 DAPs, mostly linked to AD and vascular pathology (Figure 1a‐b). Interestingly, some showed inverse associations between pathologies. ENO2, GOT1, VSNL1, MDH1 and *p*‐tau species were positively associated with AD (0.88<βstd<5.96, *p* <0.005) and negatively with WML (‐1.23>βstd>‐1.92, *p* <0.04; Figure 1a‐c). Sixteen were replicated in plasma, mostly linked to AD (Figure 1d).

64 DAPs remained significantly associated with continuous pathological measures in the overall population and 30 in Aβ+CU/MCI individuals (Figure 2a‐b). *p*‐tau species were positively associated with Aβ‐ and tau‐PET (1.92<βstd<2.95, *p* <0.0001) and negatively with WML (‐1.01>βstd>‐1.01, *p* <0.0001).

Along AD pseudotime, SMOC1 increased early and plateaued, while Aβ42 declined sharply before plateauing. UCHL1, VSNL1 and MAPT showed linear increases, while MSLN decreased progressively (Figure 2c). NPTX1, NPTX2, and TNFRSF18 decreased with higher WML load, while other DAPs related to extracellular matrix remodeling increased before plateauing (Figure 2d).

**Conclusion:**

The NULISA platform identified distinct DAPs for AD, synuclein, and vascular pathology. These had minimal overlap and significant dynamic changes along the disease progression and load which highlights the potential of proteomics panels for differentiating co‐pathologies in neurodegenerative diseases.